# Establishment of a novel platform cell line for efficient and precise evaluation of T cell receptor functional avidity

**DOI:** 10.18632/oncotarget.26139

**Published:** 2018-09-25

**Authors:** Soyoko Morimoto, Fumihiro Fujiki, Kenta Kondo, Hiroko Nakajima, Yoshiki Kobayashi, Miki Inatome, Nao Aoyama, Yuya Nishida, Akihiro Tsuboi, Yoshihiro Oka, Sumiyuki Nishida, Jun Nakata, Naoki Hosen, Yusuke Oji, Haruo Sugiyama

**Affiliations:** ^1^ Department of Cancer Immunotherapy, Osaka University Graduate School of Medicine, Osaka, Japan; ^2^ Department of Cancer Immunology, Osaka University Graduate School of Medicine, Osaka, Japan; ^3^ Department of Functional Diagnostic Science, Osaka University Graduate School of Medicine, Osaka, Japan; ^4^ Department of Cancer Stem Cell Biology, Osaka University Graduate School of Medicine, Osaka, Japan; ^5^ Department of Respiratory Medicine and Clinical Immunology, Osaka University Graduate School of Medicine, Osaka, Japan; ^6^ Department of Immunopathology, Immunology Frontier Research Center (World Premier International Research Center), Osaka University, Osaka, Japan

**Keywords:** WT1, TCR, TCR functional avidity, TCR-engineered T-cell therapy

## Abstract

Adoptive T-cell therapy with T cell receptor (TCR) -engineered T cells is an attractive strategy for cancer treatment and the success in this therapy is dependent on the functional avidity of the transduced TCRs against targeted tumor antigens. Therefore, the establishment of the methodology of the efficient and precise evaluation of TCR functional avidity has been awaited. Here, we show a novel platform cell line, named 2D3, which enables the functional avidity of transduced TCRs to be evaluated efficiently and precisely. In the 2D3, the precise TCR functional avidity of transduced TCRs is easily evaluable by the expression of green fluorescent protein (GFP) reporter gene driven by nuclear factor of activated T cells (NFAT) activation via TCR signaling. Four different TCRs of HLA-A^*^24:02-restricted Wilms’ tumor gene 1 (WT1)-specific CD8^+^ cytotoxic T lymphocytes (CTLs) were transduced into 2D3 cells and the functional avidities of these four TCRs were evaluated. The evaluated functional avidity of these TCRs positively correlated with cell proliferation, cytokine production, and WT1-specific cytotoxicity of the TCR-transduced CD8^+^ T cells in response to WT1 antigen. These results showed that 2D3 cell line was a novel and stable tool useful for the efficient and precise evaluation of the functional avidity of isolated and transduced TCRs in developing TCR-based immunotherapy.

## INTRODUCTION

Adoptive immunotherapy using tumor-associated antigen (TAA)-specific CD8^+^ cytotoxic T lymphocytes (CTLs) and/or CD4^+^ helper T (Th) cells can induce the regression of large established tumor in not only mouse models but also cancer patients [1–3]. These preclinical and clinical evidences encourage us to develop T-cell adoptive immunotherapy using genetically engineered T cells that are transduced with a T-cell receptor (TCR) gene specific for TAA. Furthermore, more recent studies have demonstrated that neo-antigens, which are generated from passenger mutations, would be promising targets for the engineered TCR-T cell therapy [4, 5]. In parallel with seeking for good targets from TAAs and neo-antigens by genome-wide approaches [6–8], novel methods analyzing huge number of TCR repertoire [9, 10] and efficiently isolating TCR gene from a single-cell [11] have been developed. Unfortunately, not all isolated TCRs can sufficiently elicit anti-tumor immunity. Hence, development of a new method for the precise and efficient evaluation of the isolated TCRs has been awaited for the prediction of clinical response in the engineered TCR-T cell adoptive therapy.

TCR affinity, TCR avidity, and functional avidity are known as an indicator to predict the *in vitro*/*in vivo* properties and behavior of the TCR-transduced T cells [12–14]. TCR affinity, which is defined as the binding-strength of TCR molecules to peptide-major histocompatibility complex (pMHC), is often used for this prediction beyond TCR’s specificity because it can standardize the strength of TCR binding to pMHC by using a numerical value (ie, K_D_ value). However, purified soluble TCR α/β complex is needed for calculating TCR affinity. It is, therefore, not feasible for screening a large number of candidate TCRs. In addition, it has been shown that TCR affinity is sometimes not consistent with actual T cell function [12, 14]. On the other hand, both TCR avidity (which is usually measured by pMHC tetramers) and functional avidity (which is assessed using a titrated concentration of antigen peptide with antigen-presenting cells) are correlated with *in vitro* cytotoxicity and *in vivo* anti-tumor activity in TCR-transduced T cells [12, 15]. Since preparation of large sets of tetramer for candidate TCRs is difficult in terms of cost, time, and effort, assessment of functional avidity must be the most adequate and feasible approach for screening of TCRs capable of provoking a good clinical response in engineered T-cell adoptive immunotherapy.

Functional avidity is assessed by phosphorylation of linker for activation of T cells (LAT) and extra-cellular signal-regulated kinase (ERK), calcium influx, and cytokine release after the stimulation with a titrated concentration of antigen peptide. Compared to TCR affinity, functional avidity is a relative indicator and easily influenced by various factors such as CD8/CD4 co-receptors and TCR clustering (ie, quantity of TCR/CD3 molecules and where and how TCR-pMHC interaction are formed) [13, 16]. Therefore, the use of primary T cells for the assessment of precise functional avidity is inappropriate because they are heterogeneous and express endogenous TCRs that cause incorrect TCR clustering by mispairing with transduced TCRs [17] and competing for CD3 molecules [18].

In this study, we describe a novel platform cell line, named 2D3, for efficient and precise evaluation of TCR functional avidity. 2D3 cells are endogenous TCR-null and CD8-positive and can express green fluorescent protein (GFP) through transcription factor nuclear factor of activated T cells (NFAT) that is activated by TCR signaling. Therefore, the establishment of 2D3 cells enabled us to selectively analyze the functional avidity of appropriately transduced TCRs by using GFP expression as a marker.

Thus, 2D3 cell line should be a good tool useful for the evaluation of the functional avidity of isolated and transduced TCRs and prediction of the TCR-transduced T cell function in developing effective adoptive T-cell immunotherapy against cancer.

## RESULTS

### Establishment of 2D3 cell line by the transduction of hCD8 and NFAT-GFP reporter genes

**Figure 1:**
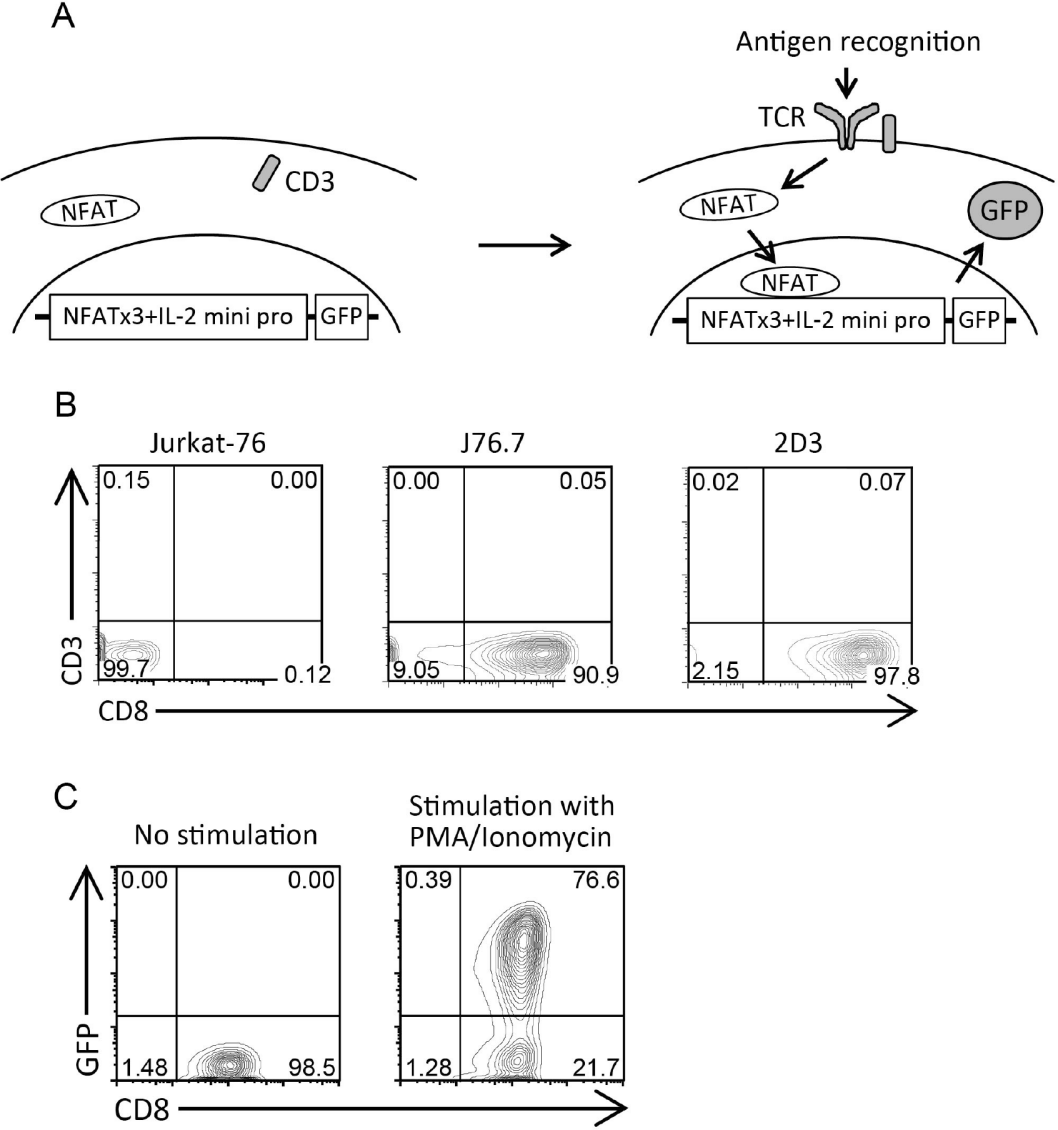
Establishment of 2D3 cell line (**A**) Schema of 2D3 cells. The transduction of TCRs into 2D3 cells recruits CD3 onto the cell surface, and appropriate TCR signaling induced by antigen recognition activates the NFAT-GFP reporter gene, followed by GFP production. NFATx3, NFAT-binding sites x3; IL-2 mini pro, IL-2 minimal promoter. (**B**) Expression of CD3 and CD8 in Jurkat-76, J76.7, and 2D3 cells. Representative contour plots are shown. (**C**) GFP expression in 2D3 cells after PMA/Ionomycin stimulation. Representative contour plots are shown.

We established a 2D3 cells in which the signals from transduced TCRs activated the NFAT, followed by the GFP expression as a selection marker for appropriately TCR-transduced cells (Figure 1A). Jurkat-76, a TCR α/β-negative sub-line of Jurkat (CD8^−^ T lymphoma cell line) was thought to be an ideal candidate as a source of the platform cell line because it could not produce endogenous TCRs and thus because transduced TCRs would be well expressed without competition with endogenous TCRs. Therefore, we transduced Jurkat-76 cells with hCD8 gene and established J76.7 cell line, and finally established CD8^+^ 2D3 cell line by the transducing the J76.7 cells with NFAT-GFP reporter gene. 2D3 cells did not express CD3 molecules on the cell surface because of lack of their endogenous TCR expression (Figure 1B), and strongly expressed GFP in the majority of cells when they were stimulated with Phorbol 12-myristate 13-acetate (PMA)/Ionomycin to activate NFAT (Figure 1C). Both expression of hCD8 and NFAT-GFP reporter genes was stable and long-lasting (data not shown). Thus, we succeeded in the establishment of 2D3 cell line suitable for evaluating the expression and function of CTL-derived TCRs.

### 2D3 is a platform cell line for efficient and precise evaluation of the expression and function of transduced TCRs

**Figure 2:**
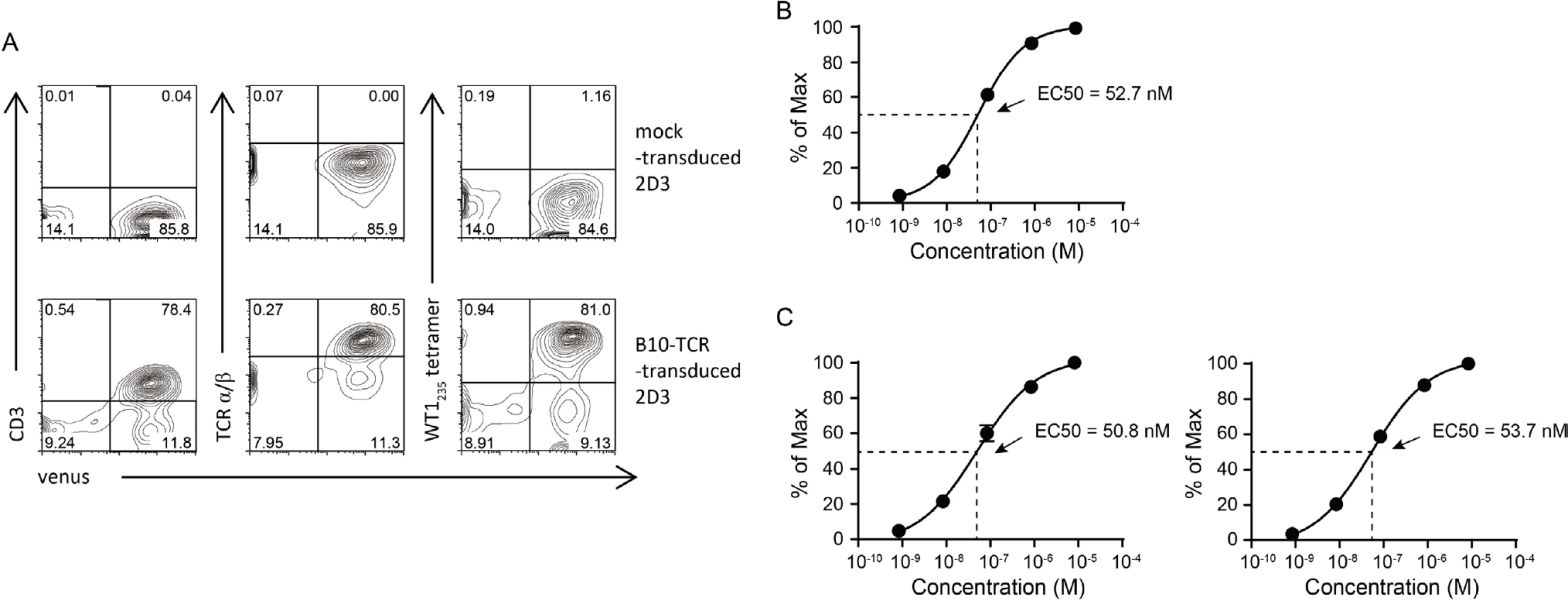
Evaluation of TCR functional avidity by 2D3 cells (**A**) Expression of CD3 and TCR in B10-TCR-transduced 2D3 cells. The 2D3 cells were stained with anti-CD3, anti-TCR α/β mAbs, and WT1_235_ tetramer. Representative contour plots are shown. (**B**, **C**) Peptide concentration-response curves in 2D3 cells transduced with three different B10-TCR constructs. The 2D3 cells were stimulated with titrated concentration of modified WT1_235_ peptide (mWT1_235_). Y-axis represents the frequency (% of max) of GFP-positive cells in 2D3 cells. (B) Peptide concentration-response curve of original α-p2A-β B10-TCR-transduced 2D3 cells. (C) Peptide concentration-response curves of codon-optimized α-p2A-β (left) and β-p2A-α (right) B10-TCR-transduced 2D3 cells. All data are mean value ± SEM (*n* = 3). All data are normalized as a percent of maximal frequency of GFP-positive cells.

To confirm that 2D3 cell line could be a platform cell line suitable for the evaluation of the expression and function of transduced TCRs, 2D3 cells were transduced with lentiviral vector encoding B10-TCR, which was the TCR that was isolated and cloned from an HLA-A^*^24:02-restricted, WT1_235_ peptide-specific CTL clone, B10 [19]. B10-TCR- and mock-transduced 2D3 cells could be monitored by the expression of Venus fluorescent protein. As expected, B10-TCR-transduced 2D3 cells expressed both CD3 and TCR α/β molecules on their surface and were WT1_235_ tetramer-positive. In contrast, mock-transduced 2D3 cells expressed neither CD3 molecules nor B10-TCR (Figure 2A). Furthermore, B10-TCR-transduced 2D3 cells showed GFP expression in WT1_235_ peptide-concentration dependent manner (Figure 2B). This WT1_235_ peptide concentration-response curve showed that Effective concentration 50 (EC50), which was often used to describe the functional avidity of TCR, was 52.7 nM (95% confidence interval (CI), 42.3–64.8 nM) for B10-TCR. In general, since TCR functional avidity is determined by several factors such as TCR affinity and quantities of TCR, CD3, and CD8/CD4 molecules, it is easily influenced by cell types used for experiments, especially by TCR constructs that regulate expression efficacy [20]. Therefore, to confirm that B10-TCR functional avidity was stably evaluable by the 2D3 cells regardless of the difference in B10-TCR constructs, we examined the response of 2D3 cells transduced with codon-optimized α-p2A-β or β-p2A-α B10-TCR that was different from original B10-TCR construct but specific to WT1_235_ peptide (Figure 2C). As expected, EC50 was 50.8 nM and 53.7 nM for codon-optimized α-p2A-β and β-p2A-α B10-TCRs, respectively, and similar to that obtained from original B10-TCR construct (α-p2A-β)-transduced 2D3 cells (Figure 2C). Thus, 2D3 cell line was thought to be a platform cell line suitable for efficient and precise evaluation of the expression and function of transduced TCRs.

### TCR functional avidity evaluated by 2D3 cells correlate with effector function of the TCR-transduced CD8^+^ T cells

**Figure 3:**
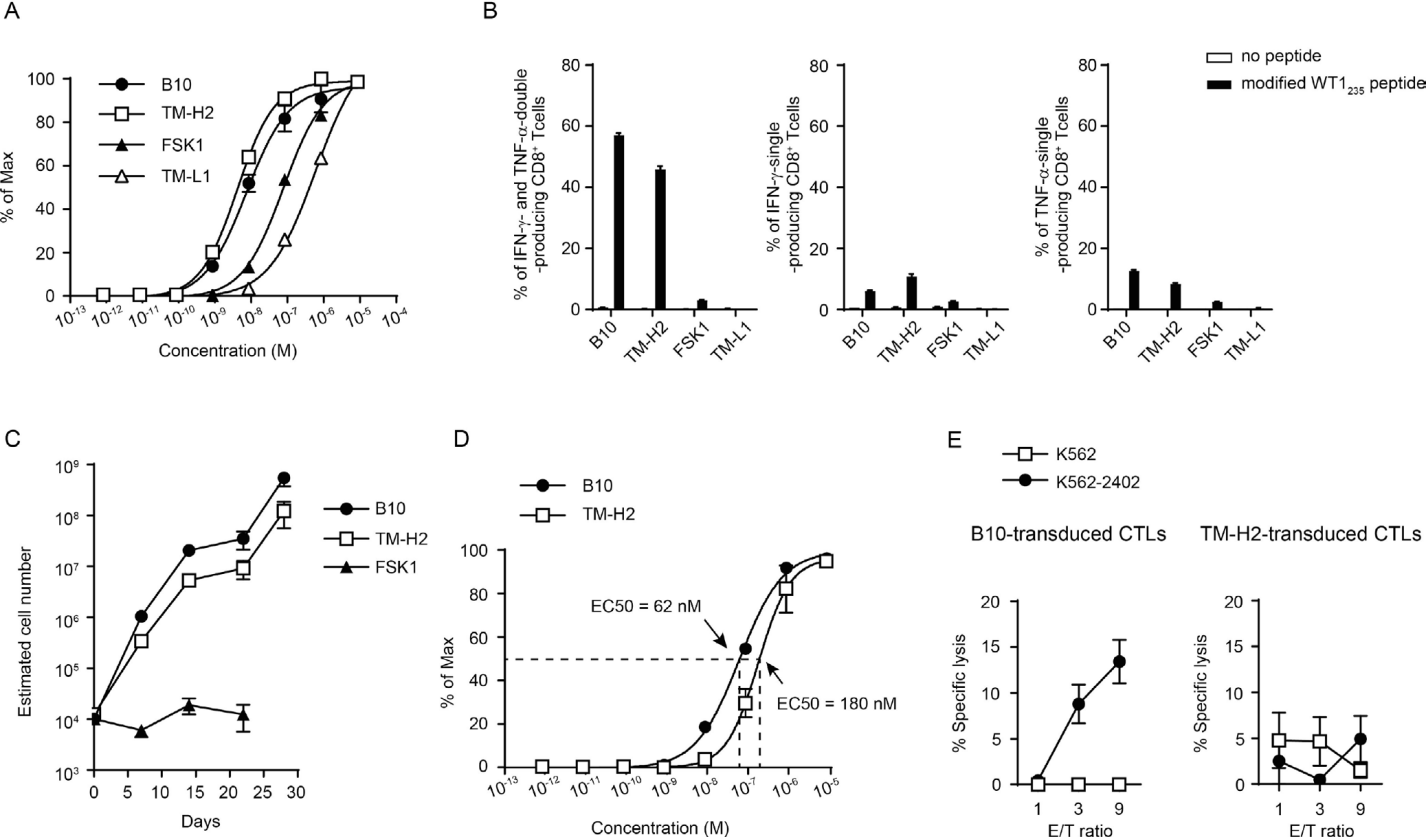
Correlation between TCR functional avidity and effector functions in the TCR-transduced CD8^+^ T cells (**A**) Peptide concentration-response curves in 2D3 cells transduced with four different mWT1_235_-specific TCRs (B10-, TM-H2-, FSK1-, and TM-L1-TCRs) for the stimulation with titrated concentration of mWT1_235_. Y-axis represents the frequency (% of max) of GFP-positive cells in 2D3 cells. (**B**) Cytokine production of mWT1_235_-specific TCR-transduced CD8^+^ T cells stimulated with 1 μg/ml of mWT1_235_. Frequencies of IFN-γ-single-, TNF-α-single-, and IFN-γ and TNF-α-double-producing CD8^+^ T cells are shown. (**C**) Proliferation of WT1-specific TCR-transduced CD8^+^ T cells. CD8^+^ T cells (5 × 10^5^ cells) were weekly stimulated with mWT1_235_, and the estimated number of the WT1-specific TCR-transduced CD8^+^ T cells was calculated every week. All data are mean values ± SEM (*n* = 3). (**D**) Peptide concentration-response curves in 2D3 cells transduced with mWT1_235_-specific B10- or TM-H2-TCR for the stimulation with titrated concentration of nWT1_235._ It should be noted that peptide used for making the peptide concentration-response curve was mWT1_235_ for (A) and nWT1_235_ for (D). (**E**) Cytotoxic activity of B10-TCR or TM-H2-TCR-transduced CD8^+^ T cells against WT1-expressing leukemic cells. Assay for cytotoxic activity was performed repeatedly and the representative results are shown. All data are replicate measurements and represent mean values ± SEM.

It is well-known that the strength of TCR functional avidity effect the proliferation, cytokine production, and cytotoxicity of TCR-transduced CD8^+^ T cells. Hence, we investigated whether the TCR functional avidity evaluated by 2D3 cells correlated with the function of the TCR-transduced CD8^+^ T cells. We established four different HLA-A^*^24:02-restricted, modified WT1_235_ peptide (mWT1_235_)-specific CD8^+^ T cell clones, isolated the TCRs from the clones, and transduced the 2D3 cells with the individual TCRs (Table 1). Four mWT1_235_-specific TCR-transduced 2D3 cells expressed GFP in response to mWT1_235_ and showed individually unique peptide concentration-response curves (Figure 3A). EC50s obtained from mWT1_235_ concentration-response curves were 43.3 nM (95% CI, 29.0–65.0 nM) for B10-TCR, 29.3 nM (95% CI, 22.1–38.9 nM) for TM-H2-TCR, 976 nM (95% CI, 815–1188 nM) for FSK1-TCR, and 1776 nM (95% CI, 1166–3019 nM) for TM-L1-TCR. Since big difference in EC50 was observed among four mWT1_235_ -specific TCR-transduced 2D3 cells, we classified these four TCRs into two groups: high-avidity TCRs (B10- and TM-H2-TCRs) and low-avidity TCRs (FSK1- and TM-L1-TCRs). Next, these four TCRs were transduced into freshly isolated CD8^+^ T cells. High-avidity TCR (B10- and TM-H2-TCRs)-transduced CD8^+^ T cells produced cytokine at much higher frequencies in response to mWT1_235_, compared to low-avidity TCR (FSK1- and TM-L1-TCRs)-transduced CD8^+^ T cells (Figure 3B). It appeared TM-L1-TCR-transduced CD8^+^ T cells rarely produced cytokine in response to mWT1_235_. Furthermore, we examined cell proliferation of B10-TCR, TM-H2-TCR, and FSK1-TCR-transduced CD8^+^ T cells by weekly stimulation with mWT1_235_ (Figure 3C). B10-TCR- and TM-H2-TCR-transduced CD8^+^ T cells remarkably expanded by day 28, whereas FSK1-TCR-transduced CD8^+^ T cells could not expand regardless of the repeated stimulation with mWT1_235_. These results demonstrated that TCR functional avidity evaluated by using 2D3 cells positively correlated with effector functions of the TCR-transduced CD8^+^ T cells.

**Table 1:** Identification of WT1_235_-specific TCRs derived WT1_235_-specific clones

TCR		V region	D region	J region	CDR3 amino acid sequences																	
B10	α	TRAV27^*^01	-	TRAJ28^*^01	C	A	G	P	L	S	G	A	S	Y	Q	L	F					
	β	TRBV9^*^01	TRBD1^*^01	TRBJ2-3^*^01	C	A	S	S	L	W	G	S	T	D	T	Q	Y	F				
TM-H2	α	TRAV20^*^02	-	TRAJ52^*^01	C	A	V	R	G	G	R	A	G	G	T	S	Y	G	K	L	T	F
	β	TRBV9^*^01	TRBD2^*^01	TRBJ2-3^*^01	C	A	S	S	V	F	G	S	S	T	D	T	Q	Y	F			
FSK1	α	TRAV19^*^01	-	TRAJ26^*^01	C	A	L	S	A	A	Y	G	Q	N	F	V	F					
	β	TRBV6-5^*^01	TRBD1^*^01	TRBJ2-1^*^01	C	A	S	S	Y	G	K	G	L	Y	N	E	Q	F	F			
TM-L1	α	TRAV17^*^01	-	TRAJ43^*^01	C	A	T	D	P	G	Y	N	N	D	M	R	F					
	β	TRBV20-1^*^01	TRBD2^*^02	TRBJ2-2^*^01	C	S	A	R	G	Q	R	E	L	S	G	E	L	F	F			

Next, we determined that the TCR functional avidity evaluated by the 2D3 cells also correlated positively with cytotoxicity of the TCR-transduced CD8^+^ T cells against HLA-A^*^24:02-positive, WT1-expressing leukemic cells. Since the WT1-expressing leukemic cells expressed natural WT1_235_ peptide (nWT1_235_), the functional avidity of the B10-TCR- or TM-H2-TCR-transduced CD8^+^ T cells was evaluated by the 2D3 cells in response to the nWT1_235_, instead of mWT1_235_ (Figure 3D). The EC50 of B10- and TM-H2-TCRs was 62 nM and 180 nM, respectively, and that of B10-TCR was approximately three times higher than that of TM-H2-TCR. As shown in Figure 3E, B10-TCR-transduced CD8^+^ T cells could lyse HLA-A^*^24:02-positive, WT1-expressing leukemic cells, while TM-H2-TCR-transduced CD8^+^ T cells could not lyse them. These results showed the positive correlation between the TCR functional avidity evaluated by 2D3 cells and the cytotoxicity against HLA-A^*^24:02-positive, WT1-expressing leukemic cells.

Taken together, these findings indicated that TCR functional avidity evaluated by 2D3 cells was clearly and positively correlated with the effector functions such as proliferation, cytokine production, and cytotoxicity of the TCR-transduced CD8^+^ T cells regardless of whether the TCR specificity was for natural or modified WT1_235_ peptide.

## DISCUSSION

In the present study, we successfully established 2D3 cell line as a platform cell line to efficiently evaluate the function of TCRs. Actually, we demonstrated the clear correlation between TCR functional avidities evaluated by the 2D3 cells and effector functions such as cell proliferation, cytokine production, and cytotoxicity of the TCR-transduced CTLs.

To our knowledge, there is no standard method to evaluate simply the functional avidity of human TCRs. T cell lines (ex. 58α^-^β^-^, TG40, or Jurkat) and T cells are often used as a platform cell for the evaluation of functional avidities of transduced TCRs, such as cytokine production, killing activity, and phosphorylation of proteins downstream of TCR signaling [21, 22]. Here is a question-are functional avidities evaluated by using these cells? Since mouse T cell lines, 58α^-^β^-^ and TG40 express CD3 molecule, human transduced TCRs are expressed on the cell surface with mouse CD3 molecule. However, it is unknown whether the complex of human TCRs and mouse CD3 can induce normal TCR signaling. Indeed, Cohen *et al.* reported that binding stability between human TCRs and mouse CD3 differed from that between human TCRs and human CD3 [23]. Nagai *et al.* used TCR-negative Jurkat/MA cells [24] that expressed only CD8 α molecule to monitor TCR signaling [25] because CD8 α could be expressed as CD8 α/α homodimers, which could bind to MHC class I molecule, without CD8 β on the cell surface. On the other hand, it is well-known that CD8 β associates with only CD8 α and cannot be expressed alone on the cell surface. Although both CD8 α/α and CD8 α/β could express on the surface, there is difference in the binding ability to MHC class I molecules between CD8 α/α and CD8 α/β [26]. In addition, CD8 β intracellular domain promotes association of lymphocyte-specific protein kinase (Lck) and LAT with surface CD8 complexes [27]. Of course, almost all mature CD8^+^ T cells express CD8 α/β heterodimer *in vivo*. Therefore, 2D3 cell line, which expresses CD8 α/β heterodimer, is a suitable platform cell line to assess TCR functional avidity. Furthermore, since 2D3 cell line is deficient in endogenous TCR expression, only transduced TCRs can be expressed on 2D3 cell surface without mispairing with endogenous TCRs. Interestingly, it has been shown that T cell recognition of pMHC can be increased up to 50-fold after priming with the same pMHC [28]. In addition, functional avidity maturation of CTLs can occur through the change of TCR clustering of various molecules such as lipid raft, Lck, and CD3 without the selection of higher affinity TCR during early stage of viral infection [29]. In addition, the same mechanism can also induce the inability of CD8^+^ T cells for the recognition of pMHC [30]. Taken together, TCR functional avidity of human T cells is variable in response to antigen stimulation. Therefore, human T cells are not suitable for platform T cells to evaluate TCR functional avidities, whereas the 2D3 cells are convenient for the evaluation of TCR functional avidities because of its functional stabilities.

Previous studies demonstrated that TCR functional avidity determined T cell fate. It is well-known that in Th1/Th2 polarization, weak TCR signaling favors Th2 differentiation and stronger one induces Th1 differentiation [31–33]. In addition, the difference in TCR functional avidity to self-antigens also has an effect on memory/effector T cell development. Allen PM and his colleagues reported that the magnitude of secondary response in Listeria-specific T cells was determined by the strength of TCR functional avidity to self-antigen [34, 35]. Furthermore, we previously demonstrated that WT1-specific CD8^+^ T cells with high-avidity TCR to WT1 peptide easily differentiated into effector T cells in TCR-retrogenic mice [36]. However, it remains unclear how TCR avidity controls T cell responses and their fate, especially memory/effector differentiation. Since TCR-stimulation of quiescent T cells such as naïve and memory T cells induces metabolic shift from catabolic to anabolic energy production, it may be speculated that the strength of TCR avidity finely regulates the metabolic condition that determines T cell differentiation [37]. Primary human T cells are not suitable for the evaluation of the role of TCR avidity in the T cell functional differentiation because they are heterogeneous and are difficult to keep the cells viable for long term after transduction of TCR genes. On the other hand, Jurkat cell line, which is a parent cell line of 2D3 cell line, is stable to viability and can respond to TCR signals. Jurkat cells can form lipid raft [38], like primary human T cells, and increase CD3 ζ and ERK phosphorylation through cholesterol removal [39]. Therefore, it appears that Jurkat cells functionally mimic primary human T cells, and thus 2D3 cell line should be useful for the study of how TCR avidity controls T cell responses and their fate.

Since 2D3 cells has a GFP, instead of luciferase, as a reporter gene, we can easily sort the activated TCR-transduced 2D3 cells by using GFP-positivity as an indicator and examine the molecules associated with the signals from the transduced TCR.

In conclusion, we demonstrate a novel platform cell line as a useful tool to evaluate efficient and precise TCR functional avidity for developing TCR-based immunotherapy.

## MATERIALS AND METHODS

### Cell lines

Human T-cell acute leukemia cell line Jurkat-76 [40] deficient in endogenous TCR expression was kindly provided by Prof Hans J Stauss (UCL Cancer Institude, London, UK). Human chronic myelogenous leukemia cell lines, K562 and HLA-A^*^24:02 gene-transduced K562, named K562-2402 were kindly gifted from Yoshiki Akatsuka (Aichi Cancer Center Research, Aichi, Japan). Transporter associated with antigen processing (TAP)-deficient and HLA-A^*^24:02-positive cell line, T2-2402 was kindly provided by Kiyotaka Kuzushima (Aichi Cancer Center Research, Aichi, Japan). All cell lines were cultured in RPMI 1640 (Nacalai Tesque Inc., 30264-56) with 10% heat inactivated fetal bovine serum (FBS) (SIGMA, 172012–500 ML) and 1% penicillin/streptomycin (Nacalai Tesque Inc., 26253–84). Lenti-X^™^ 293T cell lines were purchased by Clontech Laboratories, Inc. (632180) and were cultured in Dulbecco’s modified Eagle’s medium (DMEM) containing 4.5 mg/ml glucose (Nacalai Tesque Inc., 08458–16) with 10% heat inactivated FBS. Human CD8^+^ T cells were isolated from peripheral blood mononuclear cells using the magnetic BD IMag Cell Separation System according to the manufacturer’s instructions (BD Biosciences Pharmingen, 557941) after written informed consent was obtained from healthy volunteers. T cells were cultured in X-VIVO™ 15 (Lonza, 04–418Q) supplemented with 10% heat inactivated human AB serum (GemCell, Gemini, BioProducts, 100–512) and interleukin (IL)-2 (Imunace35, Shionogi & Co., LTD) at appropriate concentration.

### Peptides, antibodies, and reagents

Natural WT1_235_ peptide (nWT1_235_ peptide; CMTWNQMNL) and modified WT1_235_ peptide (mWT1_235_ peptide; CYTWNQMNL) were synthesized by Sigma-Aldrich Co., LLC. and Peptide Institute, Inc., respectively. For flow cytometric analysis, the following mAbs were used: anti-CD3-eFluor 450 (UCHT1, 48-0038-42), anti-TCR α/β-Phycoerythrin (PE) (IP26, 12-9986-42), anti-Interferon (IFN)-γ-PE (4S.B3, 12-7319-82) and anti-Tumor Necrosis Factor (TNF)-α-Allophycocyanine (APC) (MAb11, 17-7349-82) purchased from eBioscience and anti-CD8-APC-Cy7 (RPA-T8, 25-0088-T025) purchased from Tonbo Biosciences. PE-labeled HLA-A^*^24:02 modified WT1_235–243_ tetramer (WT1_235_ tetramer) was purchased from MBL Co., Ltd. (TS-M014-1). RetoroNectin (TaKaRa Bio Co., T100A) and anti-CD3 mAb (OKT-3, Tonbo Biosciences, 40-0037-U500) were used at concentrations of 20 μg/ml and 2 μg/ml, respectively, to stimulate human CD8^+^ T cells. NFAT-GFP reporter plasmid [41] was kindly provided by Prof. Takashi Saito (Riken Research Center for Allergy and Immunology, Yokohama, Japan). PMA (Sigma-Aldrich, P8139-1MG) and Ionomycin (Sigma-Aldrich, I0634) were used at concentrations of 25 ng/ml and 1 μg/ml, respectively.

### Establishment of 2D3 cell line

Two hundred thousand Jurkat-76 cells were subjected to electroporation with hCD8 α-E2A-hCD8 β-encoding pcDNA3.1/Zeo (+) using the Neon transfection system (Thermo Fisher Scientific Inc., MPK5000) according to the manufacturer's guidelines. hCD8 α-E2A-hCD8 β construct was kindly provided by Prof Hans J Stauss. CD8-transduced Jurkat-76 cells were single-cell sorted into 96-well round-bottom plates and stably hCD8 α/β-expressing Jurkat-76 cell line, named J76.7 was successfully established. Two hundred thousand J76.7 cells were transduced with NFAT-GFP reporter plasmid by electroporation followed by single cell sort. A single-cell-derived cell line capable of highly expressing GFP protein only when stimulated with PMA/Ionomycin was established as 2D3 cell line.

### Cloning of full-length TCR α and TCR β chain genes from WT1-specific T cell clone and lentiviral vector construction

HLA-A^*^24:02-restricted, WT1_235_-specific B10-TCR was isolated previously [19]. FSK1-TCR, TM-H2-TCR, and TM-L1-TCR were isolated from distinct HLA-A^*^24:02-restricted WT1_235_-specific CD8^+^ T cell clones and identified as described previously [42]. In order to transduce WT1_235_-specific TCR into CD8^+^ T cells, TCRs (β-p2A-α cassettes) were codon-optimized, synthesized by GeneArt (Thermo Fisher), and cloned into CSII-EF-MCS-IRES2-Venus lentiviral vector (kindly provided from Drs Hiroyuki Miyoshi and Atsushi Miyawaki, RIKEN, Tsukuba, Japan) with shRNA constructs for endogenous TCR α/β constant regions to prevent mispairing between the transduced and endogenous TCRs [17]. Lentivirus particles were obtained from transient transfection of Lenti-X^™^ 293T cells with each TCR-encoding lentiviral vector, pCAG-HIVgp, and pCMV-VSV-G-RSV-Rev (kindly provided by Dr H Miyoshi).

### Transduction of WT1-specific TCR genes

To transduce TCR-encoding lentivirus vector into CD8^+^ T cells, CD8^+^ T cells were stimulated with RetroNectin- and OKT-3-coated 48-well plate in the presence of IL-2 (40 IU/ml). On the next day, the stimulated CD8^+^ T cells were spin-infected with lentivirus vector in the presence of polybrene (10 μg/ml, Sigma-Aldrich, H9268) at 1000 g for 2 hours at 32° C. After 6–12 hours, the culture medium was changed into fresh medium supplemented with 10% heat inactivated human AB serum and IL-2 (100 IU/ml). To establish WT1_235_-specific TCR-transduced 2D3 cells, 2D3 cells were transfected with TCR-encoding lentivirus in the presence of polybrene. Venus-positive 2D3 cells were sorted and used for NFAT-GFP reporter assay as described below.

### NFAT-GFP reporter assay

To evaluate the functional avidities of WT1_235_-specific TCRs, we used 2D3 cell line. In brief, 1 × 10^5^ T2-2402 cells and 1 × 10^5^ TCR-transduced 2D3 cells were co-cultured in the presence of titrated concentration of WT1 peptide for 6 hours. The cells were washed with Phosphate buffered saline (PBS) (Nacalai Tesque, 14249–95) with 2% FBS and then measured for the frequency of GFP-positive cells in venus-positive cells using a FACSAria instrument (BD Biosciences). Data were analyzed using FlowJo 7.6.5 software (FlowJo, LLC).

### Intracellular cytokine detection assay

One hundred thousand WT1_235_-speicifc TCR-transduced CD8^+^ T cells were co-cultured with 5 × 10^4^ T2-2402 in the presence of 10 μg/ml of Brefeldin A (Sigma-Aldrich) and 1 μg/ml of natural WT1_235_ peptide or modified WT1_235_ peptide for 4 hours. After cell surface marker staining, intracellular cytokine assay was performed as described previously [42].

### Proliferation assay

CD8^+^ T cells were transduced with WT1_235_-specific TCRs as described above, and the transduced CD8^+^ T cells (5 × 10^5^) were stimulated by the co-culture with irradiated mWT1_235_ peptide-pulsed autologous PBMCs. Seven days later, the expanded cells were harvested, counted by trypan blue, and measured for the frequency of WT1_235_-specific TCR-transduced CD8^+^ T cells using tetramer assay. Five hundred thousand cells out of the expanded cells were re-stimulated and re-expanded as described above. The series of experiments was three times repeated. The number of WT1_235_-specific TCR-transduced CD8^+^ T cells was calculated as the number of venus^+^ WT1_235_ tetramer^+^ cells accumulated.

### ^51^Cr release assay

^51^Cr release assays were performed as previously described [19].

### Statistical analysis

The statistical analysis and the calculation of EC50 values were performed with GraphPad Prism 7 (GraphPad Prism Software, San Diego, CA, USA).

## Abbreviations

TCR: T-cell receptor
GFP: green fluorescent protein
NFAT: nuclear factor of activated T cells
HLA: human leukocyte antigen
WT1: Wilms’ tumor gene 1
CTL: cytotoxic T lymphocyte
TAA: tumor-associated antigen
Th cells: CD4^+^ helper T cells
MHC: major histocompatibility complex
LAT: linker for activation of T cells
ERK: extra-cellular signal-regulated kinase
TAP: transporter associated with antigen processing
FBS: fetal bovine serum
DMEM: Dulbecco’s modified Eagle’s medium
IL-2: interleukin 2
PE: Phycoerythrin
IFN: Interferon
TNF: Tumor Necrosis Factor
APC: Allophycocyanine
PMA: phorbol 12-myristate 13-acetate
EC50: Effective concentration 50
CI: confidence interval
Lck: lymphocyte-specific protein kinase
